# Efficient or Fair? Operationalizing Ethical Principles in Flood Risk Management: A Case Study on the Dutch‐German Rhine

**DOI:** 10.1111/risa.13527

**Published:** 2020-06-11

**Authors:** Alessio Ciullo, Jan H. Kwakkel, Karin M. De Bruijn, Neelke Doorn, Frans Klijn

**Affiliations:** ^1^ Institute of Geography, University of Bern Bern Switzerland; ^2^ Oeschger Centre for Climate Change Research University of Bern Bern Switzerland; ^3^ Faculty of Technology, Policy and Management Delft University of Technology Delft The Netherlands; ^4^ Deltares Delft The Netherlands

**Keywords:** Equity, flood risk management, large‐scale systems analysis

## Abstract

Flood risk management decisions in many countries are based on decision‐support frameworks which rely on cost‐benefit analyses. Such frameworks are seldom informative about the geographical distribution of risk, raising questions on the fairness of the proposed policies. In the present work, we propose a new decision criterion that accounts for the distribution of risk reduction and apply it to support flood risk management decisions on a transboundary stretch of the Rhine River. Three types of interventions are considered: embankment heightening, making Room for the River, and changing the discharge distribution of the river branches. The analysis involves solving a flood risk management problem according to four alternative formulations, based on different ethical principles. Formulations based on cost optimization lead to very poor performances in some areas for the sake of reducing the overall aggregated costs. Formulations that also include equity criteria have different results depending on how these are defined. When *risk reduction* is distributed equally, very poor economic performance is achieved. When *risk* is distributed equally, results are in line with formulations based on cost optimization, while a fairer risk distribution is achieved. Risk reduction measures also differ, with the cost optimization approach strongly favoring the leverage of changing the discharge distribution and the alternative formulations spending more on embankment heightening and Room for the River, to rebalance inequalities in risk levels. The proposed method advances risk‐based decision‐making by allowing to consider risk distribution aspects and their impacts on the choice of risk reduction measures.

## 1 INTRODUCTION

In 2002, the Elbe River, in Germany, was hit by a severe flood. The federal states of Saxony (upstream) and Saxony‐Anhalt (downstream) incurred about 8.70 and 1.75 million euros of losses, respectively. As a response, Saxony invested in flood protection measures. A decade later, in June 2013, the Elbe River was hit again by one of the most severe floods in decades (Schröter, Kunz, Elmer, Mühr, & Merz, 2015), which this time the newly reinforced embankments of Saxony could withstand. Losses amounted to about 1.19 million euros for Saxony and 1.92 million euros for Saxony‐Anhalt (higher than those previously experienced) part of which is likely to be attributed to the increased protection level upstream (Thieken et al., 2016). This raised public concern about the fairness of the implemented measures. With the aim of limiting controversies of this kind, the EU Floods Directive 2007/60/EC provides guidelines to European Member States on flood risk assessment and management. One of its key guidelines relates to the so‐called *solidarity principle*, according to which “measures are jointly decided for the common benefit” and “flood risk management plans […] shall not include measures which significantly increase risk upstream and downstream” (Directive 2007/60/EC, 2007).

The implementation of the EU Directive brings three main challenges. First, a whole system perspective must be adopted. Recently, Vorogushyn et al. (2017) called for an approach to flood risk management that accounts for interactions between atmosphere, catchments, river‐floodplain, and socioeconomic processes. Second, and connected to the former point, upstream‐downstream trade‐offs and hydraulic interactions, that is, effects of embankment breaches and cascading flooding between neighbouring protected areas, must be explicitly accounted for. Previous studies (Apel, Merz, & Thieken, 2009; Courage, Vrouwenvelder, van Mierlo, & Schweckendiek, 2013; De Bruijn, Diermanse, Van Der Doef, & Klijn, 2016; Vorogushyn, Lindenschmidt, Kreibich, Apel, & Merz, 2012) showed that neglecting hydraulic interactions leads to unreliable flood risk estimates, with risk being either overestimated or underestimated. Third, a thorough analysis of the fairness of the geographical distribution of flood risk is needed when deciding upon measures. Addressing this is not trivial, since defining what is meant by a fair risk distribution is a research problem in its own right. To illustrate the problem of what it means for a risk distribution to be fair, Hayenhjelm (2012) exemplifies two policies, which we slightly adjust to better fit the context of flood risk management. Assuming there are three areas A, B, and C, from upstream to downstream, each having an initial flood risk level of 10^6^ euros. There are two possible flood risk reduction policies, each requiring the same investments:

- ***Policy 1***: areas A, B, and C benefit of the same risk reduction of 1 × 10^5^ euros. The final risks are equal to 9 × 10^5^ euros each, which amounts to a total risk of 2.7 × 10^6^ euros.
- ***Policy 2***: areas A and B benefit of the same risk reduction of 4 × 10^5^ euros, while area C has its risk increased. The final risks are equal to 6 × 10^5^, 6 × 10^5^, and 1.3 × 10^6^ euros for A, B, and C respectively, which amounts to a total risk of 2.5 × 10^6^ euros.

Policy 1 allocates funds in such a way that every area gains the same benefit from it. In contrast, Policy 2 makes more efficient use of those funds, since the overall risk is lower. This, however, comes at the expense of area C. Which of the two policies is fairer? A policy‐maker favouring economic efficiency would deem Policy 2 as fairer, as it brings a greater risk reduction for society as a whole. Another policy‐maker might consider Policy 1 fairer, as it brings an equal risk reduction to all. And would the latter change her mind if areas A and B had started from a higher initial risk than area C? There is no unique answer to the question on what a *fair* risk distribution is. Yet, it is paramount to make the evaluation of the risk distribution an inherent part of the methods that are being applied to support large‐scale flood risk management planning.

Current decision‐support methods heavily rely on cost‐benefit analysis (Kind, 2014), which strives for maximizing the overall aggregated benefit and often neglect risk distribution considerations (Hansson, 2007). Johnson, Penning‐Rowsell, and Parker (2007) find that when funding for flood protection is allocated relying on cost‐benefit analysis, resources are not be targeted to reduce risk of the most vulnerable living in areas where the (low) exposed value does not justify large investments. To address this issue, there have been attempts to improve cost‐benefit analyses by applying distributional weights to the aggregation of benefits and costs (Kind, Botzen, & Aerts, 2017) in order to value the worse‐off more. Similarly, Adler (2011) proposes the use of a continuous prioritarian social‐welfare function for transforming peoples’ preferences for a project into a measure of overall social welfare. The proposed function is an increasing and convex function, which thus gives more importance to the marginal increase in well‐being of those with lower initial well‐being levels. Although these approaches do consider risk distribution, they are subject to two, interrelated, limitations. First, risk distribution is not a policy objective per se, as policies are ranked based on the optimization of total costs or social welfare, which thus remain the only policy objective. Second, the aggregation of all benefits into a single objective leads to a loss of information as it can hide important trade‐offs and thus adversely bias risk‐based decision support (Kasprzyk, Reed, & Hadka, 2016). To address the former limitation, a decision criterion which allows accounting for risk distribution needs to be defined. As for the latter limitation, Many‐Objective Evolutionary Algorithms (MOEAs) are typically used (Coello Coello, Lamont, & Van Veldhuizen, 2007).

MOAEs allow identifying management strategies by optimizing the system under study while balancing many conflicting criteria. Quinn, Reed, Giuliani, and Castelletti (2017) introduced the concept of rival framings, where MOAEs are used to explore the influence of alternative policy problem formulations on the policy outcomes. This approach is particularly relevant when alternative theoretical frameworks are available for addressing the same policy problem, like assessing *fairness* in risk distribution as discussed above. Previous flood risk management studies adopting MOEAs focused on either optimizing overall costs and expected risk separately (Garner & Keller, 2018; Woodward, Gouldby, Kapelan, & Hames, 2014; Woodward, Kapelan, & Gouldby, 2014) or total costs (i.e., summing costs and expected risk) for different geographical areas (Ciullo, de Bruijn, Kwakkel, & Klijn, 2019). To our knowledge, formulations where geographical risk distribution as such is considered as a policy objective to be optimized have never been explored.

The present study proposes a new decision criterion that accounts for the geographical distribution of risk and uses MOEAs to optimize both total costs *and* equity in risk distribution. The aim is to explore the policy implications of adopting alternative ethical principles in the way fairness is conceptualized and operationalized. We do so by solving a flood risk management problem according to *four* alternative *problem formulations*, that is, the policy problem to be solved, each corresponding to a different way of operationalizing fairness. Although the study is primarily methodological in character, we develop it on a case study of the transboundary area of the German‐Dutch Lower Rhine River in order to connect as close as possible to a realistic and geographically differentiated flood risk situation.

After discussing alternative ethical principles and introducing the new decision criterion in section 2, we introduce the case study area in section 3, we briefly describe the simulation model, measures and outcomes in section 4, and introduce the four problem formulations in section 5. Finally, we explain the adopted method in section 6, we present the results in section 7, we discuss them and provide conclusions in section 8.

## 2 THE RISK DISTRIBUTION PROBLEM AND THE PROPOSED DECISION CRITERION

In this section, we introduce the philosophical basis of cost‐benefit analysis, we discuss the risk distribution problem and the way it has been tackled. After that, we introduce ethical theories dealing with the problem of fairly allocating risk and, finally, we introduce the new decision criterion to account for risk distribution and its inclusion in an optimization framework.

Cost‐benefit analysis is the dominant paradigm in risk policy (Hayenhjelm & Wolff, 2012). It is essentially motivated by the desire to efficiently allocate scarce economic resources. In cost‐benefit analysis, policy alternatives are deemed feasible if and only if the sum of expected benefits exceeds the sum of expected costs, with the most preferable policy being the one maximizing the net benefits (i.e., benefits minus costs).

There are three major problems with cost‐benefit analysis applied to flood risk management. First, intangible damages, including loss of human lives, need to be monetized in order to include them in the analysis (Kind, 2014). Second, in cost‐benefit analysis, risk is typically defined as the expected value of flood damage in each given year. Expected values, however, do not capture society's risk aversion, that is, the societal higher concern for rare and catastrophic flood events than for frequent, less impacting, ones (Merz, Elmer, & Thieken, 2009). Third, as Hansson (2007) points out, by aggregating costs and benefits, cost‐benefit analysis relies on the assumption of *interpersonal aggregation*, where it is assumed that one person's or group's disadvantage can be fully compensated and justified by another person's or group's advantage. It is thus acceptable to treat the involved parties in such a way that it results in an asymmetric distribution of benefits, as long as a greater benefit to society at large can be achieved (Hayenhjelm, 2012).

In response to the third issue, distributive weights may be applied in the process of aggregating costs and benefits (Kind et al., 2017). Defining distributive weights requires specifying people's utility function (Adler, 2011). Typically, the better off people are, the lower the increase in marginal utility and vice‐versa. Therefore, although *interpersonal aggregation* is still required, the use of distributive weights allows accounting for people's different levels of well‐being. It has been demonstrated that ranking policies through cost‐benefit analysis with distributive weights, in that it requires to specify people's utility function, is in fact equivalent to the use of Social Welfare Functions (SWFs) (Adler, 2011). SWFs are used to assess the social welfare of policies based on individual utilities, with the best policy being the one maximizing social welfare. Specifying the form of SWF is crucial. Adler (2011) proposes the use of continuous prioritarian SWF, that is, an increasing and strictly concave function such that there is a decreasing marginal moral value for increasing utility levels. This implies that higher weight is given to increases in utility of people with initially lower wealth. Therefore, both cost‐benefit analysis with distributive weights and SWF as defined by Adler (2011) strive for overall efficiency while accounting for interpersonal risk distribution. However, the distribution of risk is *not* a policy objective per se, as policy evaluation is solely based on the maximization of aggregated welfare. Alternative ethical theories, such as egalitarianism and prioritarianism, do, instead, require risk distribution to be the ultimate goal of policy evaluation (Lamont & Favor, 2017).

Strict egalitarianism conceives inequality as such not to be justifiable on moral grounds, thus, requiring a perfectly equal distribution of benefits. Strict egalitarianism is, however, subject to the so‐called *levelling down objection* (Gosepath, 2011; Hayenhjelm, 2012; Lamont & Favor, 2017): for the sake of equality, everybody may end up being equally worse off, which is obviously undesirable. An alternative to egalitarianism is prioritarianism, according to which benefits should be prioritized to the worst‐off (Parfit, 1997). According to prioritarianism, inequalities are justified if they benefit the worst‐off. Egalitarianism and prioritarianism differ in what they consider to be the main concern. Egalitarianism is concerned with *relative* levels, that is, benefit distribution is unfair if and only if one person is worse off than another person. Prioritarianims, instead, is concerned with *absolute* levels, that is, benefit distribution is unfair if the level of well‐being of the worse‐off is deemed unfair, regardless other people's levels. Irrespective of the reason why a distribution is deemed unfair, there is the need to complement cost‐benefit analysis goals of economic efficiency with benefit distribution goals in order to build the *pluralistic* decision framework advocated by Johnson et al. (2007) where system‐wide objectives, such as reducing aggregated costs and benefits, are achieved while ensuring that all interested parties have an equal opportunity of having their risk reduced. With this aim, we propose a decision criterion to account for the distribution of benefits between affected individuals, groups or, as applied in the present study, geographical areas.

**Fig 1:**
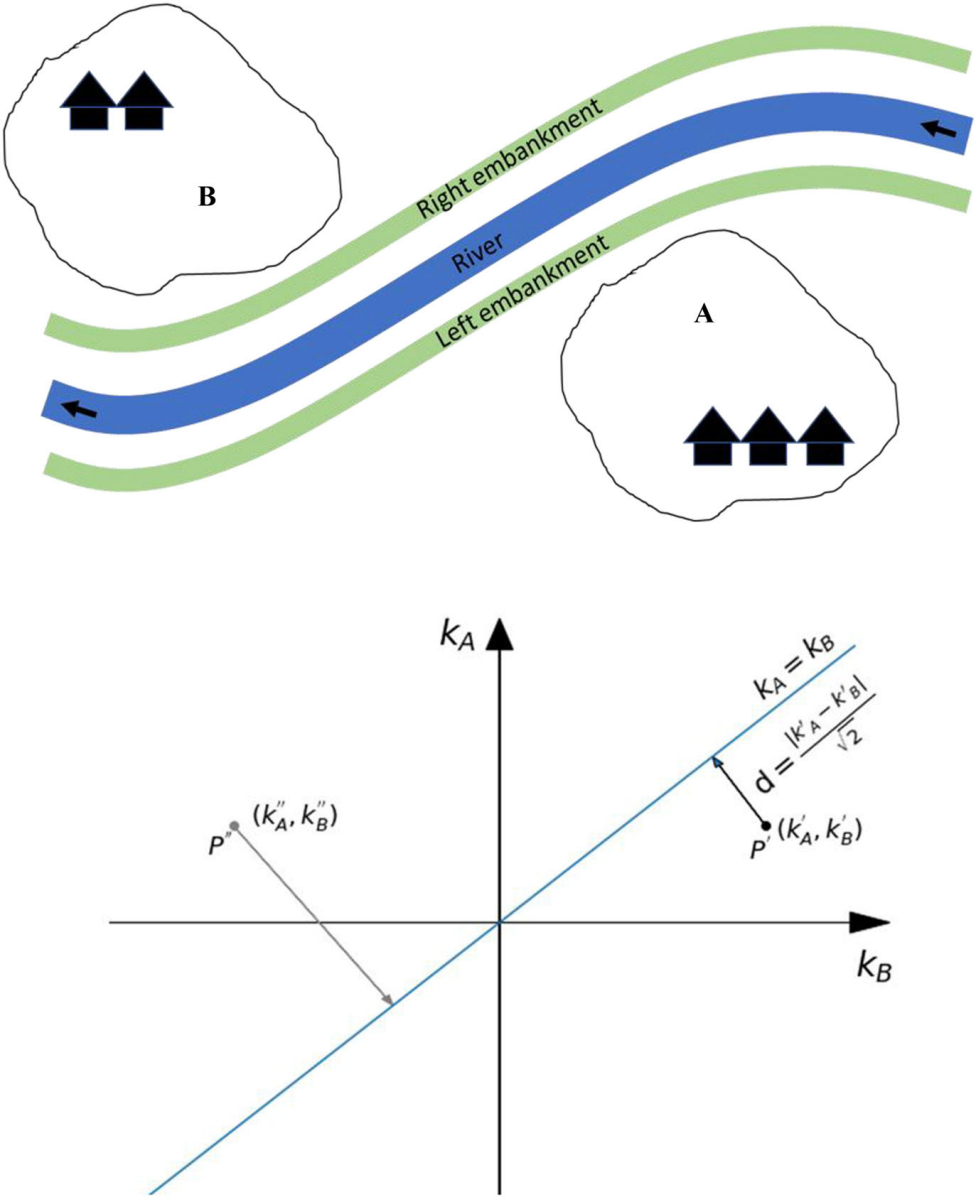
Top: stylized representation of a flood risk system with two flood protected areas, A and B. Bottom: visualization of the geometric approach used to quantify the distribution of benefits among areas. The axes represent the outcome indicator of area A, *kA*, and area B, $k_{B}$. The bisector is the line of equal benefits, that is, a condition where area A and B have the same k. Points P’ and P’’ indicate the effect of two distinct policies on the outcome indicators. The distance *d* indicates the geometric distance between a point to the bisector line, that is, *d* indicates how far the distribution of benefits brought by a given policy is from an equal distribution.

The approach is illustrated in Fig. 1, where the top figure shows a stylized flood risk system with two flood protected areas, A and B, and the bottom figure provides a visualization of the proposed criterion. In particular, the distribution of benefits is quantified by the distance *d* between the two flood‐protected areas, A and B, in terms of a generic outcome of interest *k* quantifying the benefits of a given policy (namely the difference in performance between the policy and the status quo) with its exact definition to be agreed upon based on what is the entity that ought to be distributed. The bisector is the line of equal benefits, that is, a condition where area *A* and B have the same *k*. The status quo is given by the point of origin.

As an example, we shall consider point *P*′, representing a policy with performances $k_{A}^{\prime }\;$and $k_{B}^{\prime },$ with$\;k_{B}^{\prime }>\;k_{A}^{\prime }>0$. The distance between point *P’* and the line of equal benefits is:

(1) $$d=\frac{\left|k_{A}^{\prime }-\;k_{B}^{\prime }\right|\;}{\sqrt{2}},$$

Moving from point *P*′ (where $k_{B}^{\prime }>k_{A}^{\prime }$) to the closest point on the bisector (where $k_{B}=k_{A}$) implies a *reallocation* in the performance of indicator *k* from area B to area A.

In general, the smaller the distance, the closer one is to an equal distribution of benefits. Care must be taken, however, not to incur in a situation in which, for the sake of equity, everybody is worse off. This is the case of point *P*″, where $k_{A}^{{}^{\prime \prime }}>0,\;k_{B}^{{}^{\prime \prime }}<\;0$ and minimizing the distance *d* would imply a tendency to a situation where $k_{A}^{{}^{\prime \prime }}<0,\;k_{B}^{{}^{\prime \prime }}<0$, that is, where both indicators perform worse than in the status quo. Therefore, in using this new distance criterion, we constrain the analysis to the $k_{A},\;k_{B}>0$ domain.

In light of the introduced concepts of cost‐benefit analysis, egalitarianism and prioritarianism, and using the proposed decision criterion, we define four problem formulations in section 5 for managing flood risk for the case study described in section 3.

## 3 THE CASE STUDY

**Fig 2:**
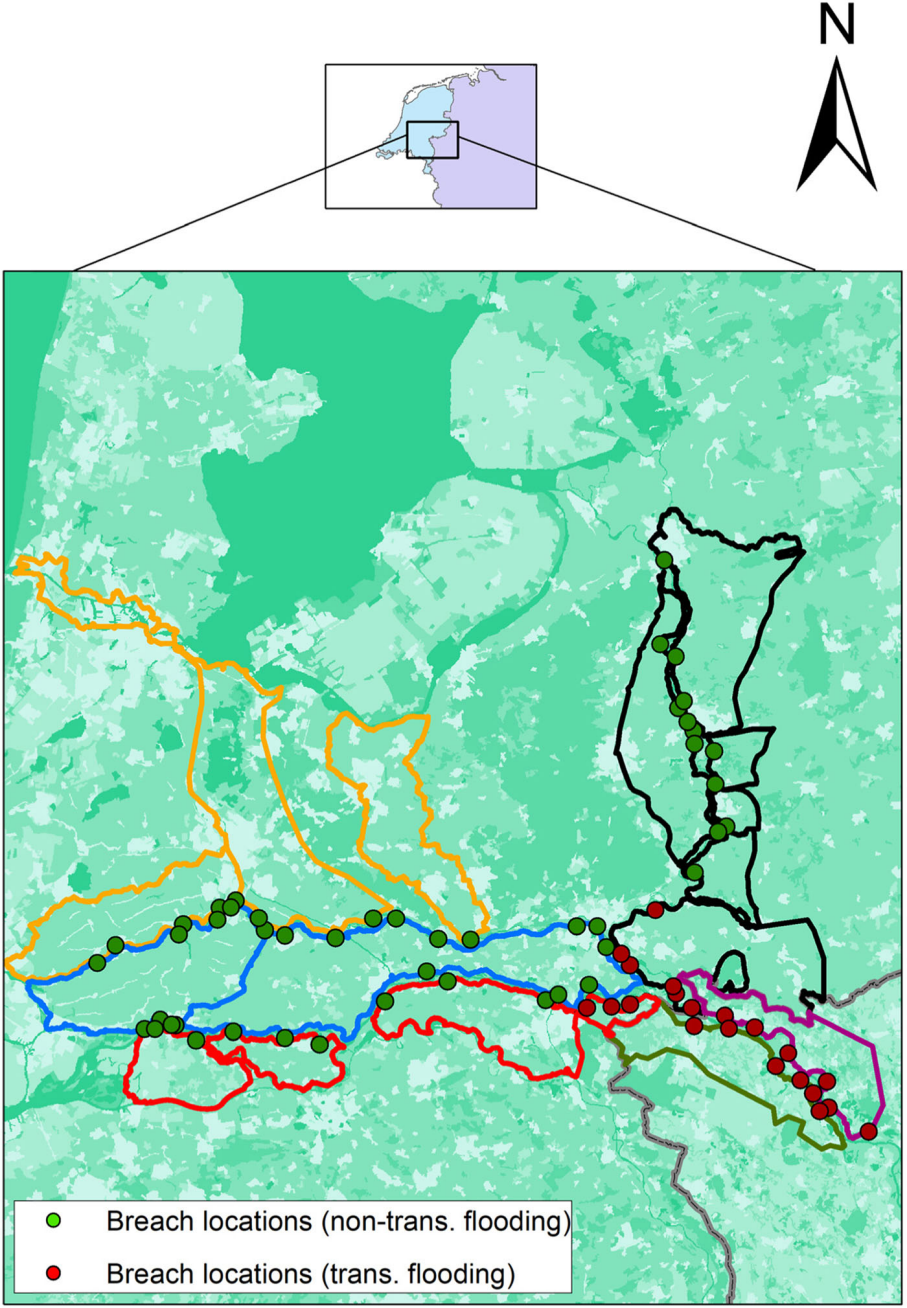
Case study area. Six macroareas of interest are identified: four Dutch areas (*area 0*, in red; *area 1*, in blue; *area 2*, in orange; *area 3*, in black) and two German areas (*area 4*, in green; *area 5*, in purple). The administrative country border is depicted in grey. Dots represent breach locations, with red dots indicating breaches leading to damage in both countries.

The present study focuses on the downstream part of the Lower Rhine, from Bislich (right bank) and Xanten (left bank) up to the end of the nontidal zone of the Dutch Rhine (Fig. 2). The Dutch Rhine bifurcates into three distributaries, the Waal River to the southwest, the Nederrijn to the west, and the IJssel River to the north. The study area thus includes parts of German and Dutch territories.

In Fig. 2, the administrative country border is depicted in grey and the thick closed lines represent the so‐called *dike‐ring areas*, that is, alluvial plains that are protected from flooding by connected embankments. Six macroareas of interest are identified based on the *dike‐ring areas* and results in the following sections will refer to these areas. The geographic names of each area are shown in Table I. Furthermore, we recognize 70 potential breach locations of interest, that is, places where the flood protection might fail resulting in flooding. Breach locations in red affect transboundary dike‐ring areas, implying that flooding causes damage in both countries, regardless of the country in which the breach is located. In fact, all considered potential breach locations in Germany result in transboundary flooding.

**Table I:** The Geographic Areas of Interest

Identifier	0	1	2	3	4	5
Name	Waal River South	Central River area	Nederrijn‐Lek North	IJssel River valley	Ooij Düffelt polder	German Rhine North

## 4 THE SIMULATION MODEL, MEASURES, AND OUTCOMES

**Fig 3:**
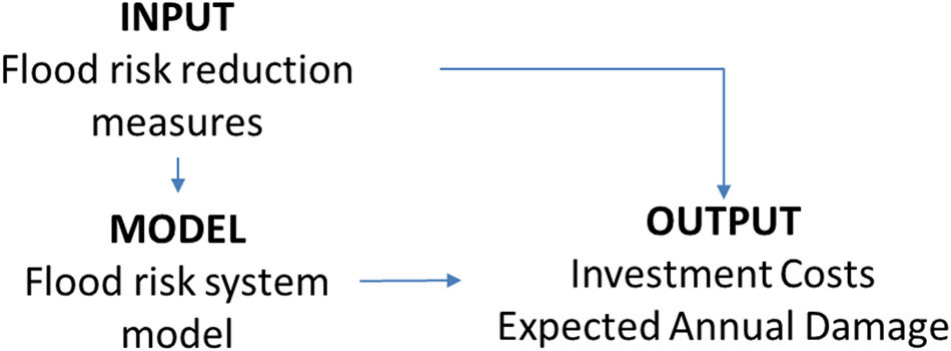
Schematic view of inputs and outputs of the simulation model. More information on the simulation model are provided in the Supporting Information and in Ciullo et al. (2019).

The proposed simulation model is a fast, integrated metamodel (Haasnoot et al., 2014). It builds upon the one introduced in Ciullo et al. (2019), which was developed for the IJssel River (approximately *area 3* in Fig. 2). However, there are two main differences. First, a more diverse set of possible flood risk management measures. In Ciullo et al. (2019), only embankment heightening was considered; the current version also includes making *Room for the River* and influencing the discharge distribution over the three river branches. Second, a damage model was developed to ensure consistency in damage assessment in both countries. We describe the above‐mentioned differences in the following sections and provide a schematic representation of the model's inputs and outputs in Fig. 3. More information about the model is provided in the Supporting Information and we refer the reader to Ciullo et al. (2019) for a detailed description.

### 4.1 Flood Risk Reduction Measures

Three flood risk reduction measures are possible: embankment heightening, making Room for the River, and changing the discharge distribution at the bifurcation points. Embankments can be raised up to 1 meter, with steps of 10 cm. Embankment raising costs are simulated as in Eijgenraam Brekelmans, den Hertog, and Roos (2017):

(2) $$I=\left\{\begin{array}{c} 0\;\;\;\;\;\mathrm{if}\;u=0\\ \left(c+bu\right)e^{-\lambda \left(W+u\right)}\;\;\;\mathrm{if}\;u>0 \end{array}\right.$$

where *u* is the degree of embankment heightening; parameters *c* and *b* are fixed and variable costs, respectively; λ is a scale parameter, and *W* is the cumulative embankment heightening over the entire planning period, establishing increasing costs per heightening unit as embankments become higher. As in the present work, a single optimal embankment height is identified, W is assumed to be equal to zero. Parameters *c*, *b*, and λ are assigned per stretch of the embankment system and their values are provided by De Grave and Baarse (2011).

As for making room for the river, there are 156 Room for the River projects available to choose from along the *Dutch Rhine*, based on an existing database (Van Schijndel, 2005). For our simulation, a project can simply be either implemented or not. Costs of Room for the River projects range from 50.000 euros to about 2 billion euros.

As for making changes to the discharge distribution, there are two bifurcation points of interest; the default flow distributions are the ones provided by a *SOBEK* model calibrated on the case study. At each bifurcation point, it is assumed that a distribution change of plus/minus 30% of the default distribution can be implemented. There are no costs associated with changing the discharge distribution as it may be accomplished by adjusting the hydraulic structures currently in place.

### 4.2 Damage Estimation

Due to the transboundary nature of the problem at hand, consistency of damage estimates between the two countries is paramount, thus, the exposure data as well as the adopted damage model should come from the same source and rely on the same assumptions.

We use exposure data from the CORINE Land Cover dataset (EEA, 2016) and the global flood depth‐damage functions proposed in Huizinga, De Moel, and Szewczyk (2017). These provide normalized damage functions per land use category per continent as well as a country‐wise maximum damage value. The final flood depth‐damage functions result from the multiplication of the two and they are thus country‐specific.

The CORINE Land Cover dataset distinguishes 44 classes while the global flood depth‐damage model provides damage functions for only a few land‐use categories: residential, commercial, industrial, agricultural, infrastructural, and transportation, referred to here as “JRC Land‐use categories.” Each CORINE class is related to percentages of JRC land‐use categories. For example, the CORINE land use class 111 consists for 50% of “residential,” for 5% of “commercial,” and for 18% of “transport.” Therefore, the final damage functions of each CORINE class result from the weighted sum of damage functions for each JRC land‐use category, with the weights being the percentage of land‐use categories in each class. The CORINE classes, the percentage of JRC land‐use category per class can be found in Huizinga (2007).

Damage is calculated based on flooding simulations from the VNK project (in Dutch: *Veiligheid Nederland in Kaart, in English FLORIS: Flood Risks and Safety in the Netherlands*) (Jongejan & Maaskant, 2015). VNK is a major flood risk analysis project which relies on flooding simulations for three flood levels: the design flood levels as well as those that are expected 10 times more frequently and 10 times less frequently. Each of the three VNK flooding simulations has a return period and a water level in the river associated with it. Consequently, for each location a relationship can be established between return periods, water levels in the river and damages. Damages of flood events with return periods other than the three simulated are found by linear interpolation.

Finally, VNK also provides a maximum water depth map per *dike‐ring area*, meaning that no higher water depths can be reasonably expected. A maximum damage per *dike‐ring area* can thus be calculated, which is used as upper boundary in case the superimposition of damage estimates of different breach locations in the same *dike ring* would exceed this maximum.

### 4.3 Model Outcomes

The model produces eight outcomes of interest, viz. the *present value of expected annual damage, EAD* in each of the six areas in Fig. 2 and the *investment costs, I* of a given policy for the two countries. The latter are the sum of the costs of heightening the embankments and those of the implemented Room for the River projects. We refer to total costs as the sum between the present value of expected annual damage and investment costs. The present value of expected annual damage is defined as follows:

(3) $$EAD\left(T,r\right)=\sum_{t=1}^{T}\frac{\int_{H_{\min}}^{+\infty }p\;\left(H\right)L\left(u,\;H\right)dH}{{\left(1+r\right)}^{t}}\;$$

where *L* is the flood damage (€); *H* is the water level in the river (m + m.s.l.), with *H*_min_ being the lowest water level causing flood damage; *u* represents the effect of the chosen policy on the loss estimates; *p(H)* is the probability density function of a given water level H; *T* is the planning period (i.e., 200 years), *r* the discount rate (3.5% per year). Clearly, lower (higher) values of discount rate increase (decrease) investments. However, using a single value suffices our scope of exploring the differences regarding where investments are directed across formulations, as the same discount rate would anyway apply to all of them.

## 5 THE PROBLEM FORMULATIONS

In this section, we introduce four alternative problem formulations for the case study, based on the theories and decision criterion introduced in section 2. Furthermore, connections of the four formulations and their underlying principles to either previous studies or established practice in flood risk management are provided.

The first and second problem formulations follow a cost‐benefit analysis approach. The third and fourth complement cost‐benefit analysis by also using the new decision criterion but they differ in their conceptualization of the outcome indicator *k*, that is, in what ought to be distributed. In the risk ethics literature, the following have been proposed as entity to be distributed: economic resources, final risk levels, and degree of risk reduction (Doorn, 2015). We focus on the latter two.

Ideally, when applying the new decision criterion, the distance is calculated between each pair of areas. However, in so doing the number of decision criteria to be optimized in a case such as the one in Fig. 2 would soon become too large. That is why in the present work the distance is calculated between each area *i* and all the other ones, as if they were a single area. Referring to the bottom panel of Fig. 1, the axes would thus represent the outcome indicator of area *i*, *k_i_*, and the aggregation of all other areas but *i*,$k_{\Sigma_{j,\;j\neq i}}$.

In all problem formulations*, investment costs I* in the Netherlands (areas from 0 to 3) are subject to a maximum investment constraint of 1 billion. In Germany (areas 4 and 5), because there are fewer locations and embankment heightening is the only possible intervention, the total maximum investment costs are still below what is practically reasonable to invest. Thus, no investment cost constraint is applied.

In the mathematical formalization of the problem formulations provided in the next subsections, indices *i* and *j* refer to the five flood protected areas in Fig. 2 and can thus take value from 0 to 5.

### 5.1 First Problem Formulation: *Cost‐Benefit Analysis (CBA)*

The first problem formulation follows a cost‐benefit approach (in the remainder, *CBA*) and it is defined as follows:

(4) $$\mathrm{minimize}\;\sum_{i=0}^{i=3}\left(I_{i}+EAD_{i}\right),\;\;\sum_{i=4}^{i=5}\left(I_{i}+EAD_{i}\right)\;\;\;$$

$$\mathrm{with}\;\;\;\sum_{i=0}^{i=3}I_{i}\leq 10^{9}\;\;\;\;\;\;\;\;$$

This formulation is equivalent to the one adopted in previous studies like those of Brekelmans, den Hertog, Roos, and Eijgenraam (2012), Kind (2014), and Eijgenraam et al. (2017).

### 5.2 Second Problem Formulation: *Constrained Cost‐Benefit Analysis (cCBA)*

The second problem formulation constrains the cost‐benefit analysis approach (in the remainder, *cCBA*) by guaranteeing that no area is worse than in the status quo. It is defined as follows:

(5) $$\mathrm{minimize}\;\sum_{i=0}^{i=3}\left(I_{i}+EAD_{i}\right),\;\sum_{i=4}^{i=5}\left(I_{i}+EAD_{i}\right)\;$$

$$\begin{array}{ccc} \mathrm{with} & & \;\;\sum_{i-0}^{i-3}I_{i}\leq 10^{9}\;\;\;\;\;\\ & & \;\;EAD_{i}\leq EAD_{0|i}\;\forall i\varepsilon \left(0,1,2,3,4,5\right) \end{array}$$

This formulation constrains cost‐benefit analysis, and thus, resembles the principles of the Dutch flood risk management policy, where differentiation of protection levels based on economic considerations is aimed for while at the same time basic security is provided to all citizens (Jonkman, Jongejan, & Maaskant, 2011; Van Der Most, 2010).

### 5.3 Third Problem Formulation: *Egalitarian*

In the third problem formulation, in addition to minimizing total costs, the distance between performance indicators *k_i_*, $k_{\Sigma_{j,\;j\neq i}}$ is minimized, with the two indicators being defined as follows:

$$\;k_{i}=\frac{\left(EAD_{0|i}-EAD_{i}\right)}{\sum_{j}\;EAD_{0|j}}\;\;\;$$

$$\;k_{\Sigma_{j,j\neq i}}=\frac{\sum_{j,j\;\neq i}\left(EAD_{0|j}-\;EAD_{j}\right)}{\sum_{j}\;EAD_{0|j}}$$

where risk reductions are normalized over the total initial risk ($\sum_{j}\;EAD_{0|j}$) for convenience. This problem formulation seeks for an *equal distribution of risk reduction* (difference between the initial risk level *EAD*_0_ and the final risk *EAD*) and it thus qualifies as an *egalitarian* problem formulation. As such, this formulation resembles the flood risk policy principles of countries which apply equal protection standards to all areas, for example, Austria (Thaler & Hartmann, 2016).

The problem formulation reads as follows:

(6) $$\mathrm{minimize}\;\sum_{i=0}^{i=3}\left(I_{i}+EAD_{i}\right),\;\sum_{i=4}^{i=5}\left(I_{i}+EAD_{i}\right)\;$$

$$\;\;\;\;\;\;\;\;\;\frac{\left|k_{i}-\;k_{\Sigma_{j,\;j\neq i}}\right|\;}{\sqrt{2}},\;\;\forall i,\;j\varepsilon \left(0,1,2,3\right)$$

$$\;\frac{\left|k_{i}-\;k_{\Sigma_{j,\;j\neq i}}\right|\;}{\sqrt{2}},\;\;\mathrm{for}\;i=4,j=5$$

$$\mathrm{with}\;\sum_{i=0}^{i=3}I_{i}\leq \;10^{9}\;\;\;\;\;$$

$$\;\;\;EAD_{i}\;\;\leq EAD_{0|i},\;\forall i\varepsilon \left(0,1,2,3,4,5\right)$$

Thus, there are seven decision objectives to be minimized. Two cost objectives and five distance objectives. With respect to the latter, four objectives concern the four areas in the Netherlands and the remaining one concerns the two German areas together.

### 5.4 Fourth Problem Formulation: *Prioritarian*

The fourth problem formulation resembles the third. The main difference is that the performance indicators *k_i_*, $k_{\Sigma_{j,\;j\neq i}}$ are now defined as:

$$k_{i}=\frac{\left(EAD_{0|i}-\;EAD_{i}\right)}{EAD_{0|i}}$$

$$\;k_{\Sigma_{j,\;j\neq i}}=\;\frac{\sum_{j,\;j\;\neq i}\;\left(EAD_{0|j}-\;EAD_{j}\right)}{\sum_{j,\;j\;\neq i}\;EAD_{0|j}}$$

In this problem formulation, an *equal distribution of relative risk reduction is sought*. The *relative risk reduction* is defined as the difference between the initial risk level $EAD_{0}$ and the final risk *EAD*, normalized by the initial risk level. This means that, in order to minimize the distance, areas with a higher initial risk will benefit from larger risk reductions. This formulation, therefore, prioritizes interventions to higher risk areas and in this it qualifies as a *prioritarian* formulation. It reads as follows:

(7) $$\mathrm{minimize}\;\sum_{i=0}^{i=3}\left(I_{i}+EAD_{i}\right),\;\sum_{i=4}^{i=5}\left(I_{i}+EAD_{i}\right)\;$$

$$\;\frac{\left|k_{i}-\;k_{\Sigma_{j,\;j\neq i}}\right|\;}{\sqrt{2}},\;\;\;\forall i,\;j\varepsilon \left(0,1,2,3\right)$$

$$\;\frac{\left|k_{i}-\;k_{\Sigma_{j,\;j\neq i}}\right|\;}{\sqrt{2}},\;\;\;\mathrm{for}\;i=4,j=5$$

$$\mathrm{with}\;\sum_{i=0}^{i=3}I_{i}\leq 10^{9}\;\;\;$$

$$\;\;EAD_{i}\leq EAD_{0|i}\;\;\forall i\varepsilon \left(0,1,2,3,4,5\right)$$

This formulation, in that it relies on the principle of prioritizing interventions to higher risk areas, resembles the flood risk management approach followed in the United Kingdom, where expenditures for flood defenses are allocated taking into account the presence of deprived areas (Penning‐Rowsell, Priest, & King, 2016).

## 6 METHODS

The four problem formulations are solved using the Many Objective Evolutionary Algorithm (MOEA) (Coello Coello et al., 2007) ε‐NSGAII (Kollat & Reed, 2005). A detailed description of ε‐NSGAII is provided in the Supporting Information. MOEAs represent metaheuristic approaches to find a Pareto‐approximate set of solutions, that is, solutions for which it is impossible to improve a single objective without decreasing the performance of at least one other objective.

In the MOEA search, expected annual damages are calculated based on 10 upstream high‐flood waves (i.e., with probabilities of less than 1:125 per year). A larger number would require longer computation times and make the optimization unfeasible, as the total number of required evaluations becomes too large. Once optimal policies are identified, however, their performance is re‐evaluated for a larger sample of 2,500 river flood waves. After that, a final set of policies is selected such that each policy (irrespective of the formulation it derives from) is Pareto dominant in terms of total costs in the two countries and expected annual damage at the six flood‐protected areas. This guarantees that no policies that after the re‐evaluation exhibit higher total costs and higher risks at all areas—and which are thus undefendable—are considered.

Finally, in order to quantify inequality, we use the Gini index. The Gini index is widely used in welfare economics in order to measure income inequality, and is defined as follows:

$$G\;=\frac{\sum_{i=1}^{n}\sum_{j=1}^{n}\;\left|x_{i}-x_{j}\right|}{2n^{2}\bar{x}}$$

where *x* is an observed value, *n* is the number of values, and $\bar{x}$ is the mean value.

The analysis is carried out through the Exploratory Modelling and Analysis Workbench (EMA‐Workbench) (Kwakkel, 2017), an open source toolkit developed in the Python programming language.

## 7 RESULTS

In what follows, we use the term policy to address a specific combination of interventions, comprising different locations and degree of (1) raising embankments, (2) making Room for the River, and (3) changes to the discharge distribution. Pareto fronts of the optimal policies resulting from the ε‐NSGAII search, the epsilon values of the decision objectives, the scores of the performance metrics, and the comparison between the performance of policies under the reference sample and the larger sample are reported in Supporting information. As explained in section 6, the analysis presented below relies on policies which, after the re‐evaluation, are Pareto dominant in terms of total costs and expected annual damages.

**Fig 4:**
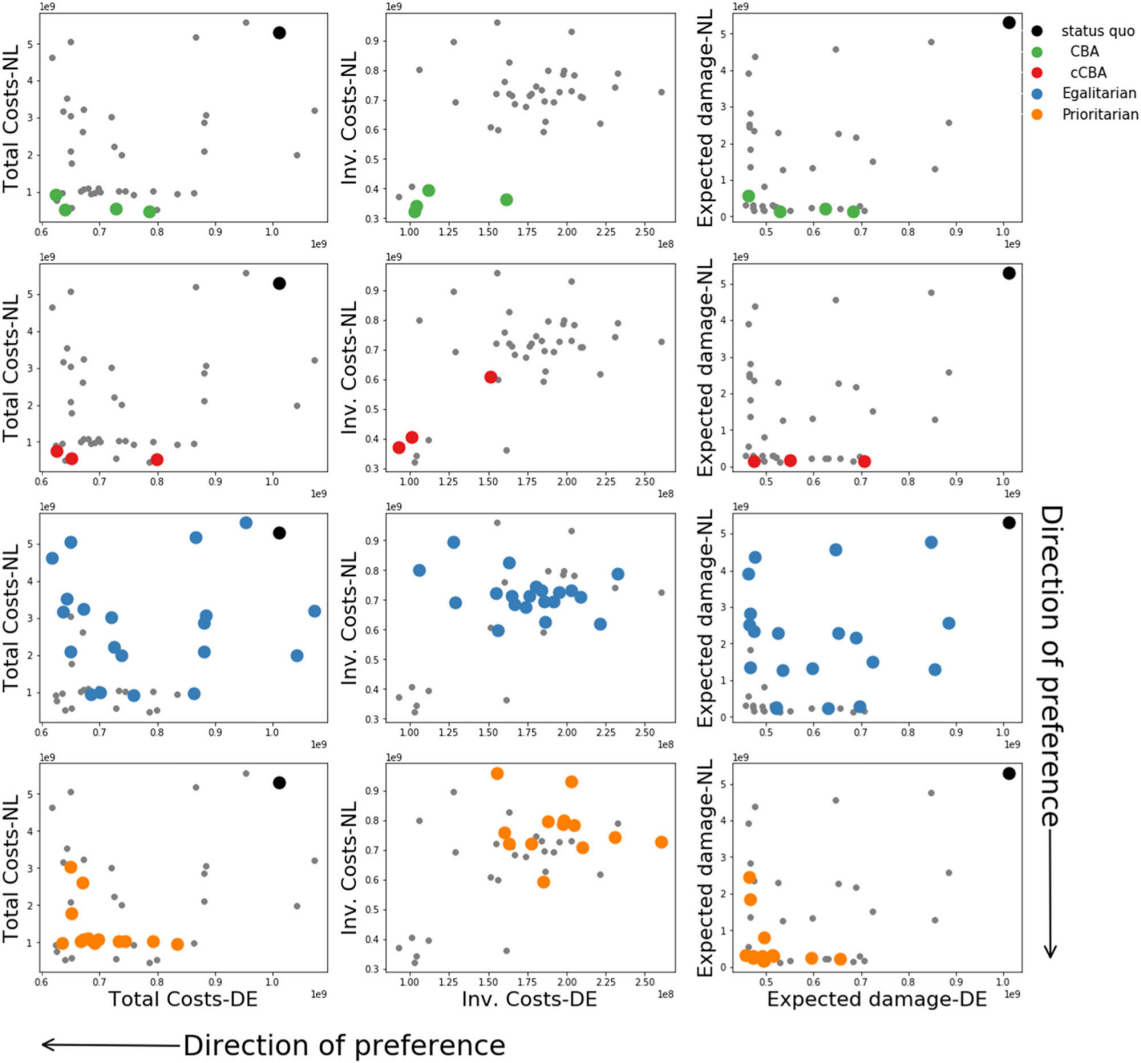
Total costs (left column), investment costs (mid‐column), and final risk (right column) of the Netherlands (*y*‐axis) and Germany (*x*‐axis) for all problem formulations, which follows the colors reported in the legend. In each box all policies, regardless the problem formulation, are plotted (grey dots) and those belonging to the problem formulation of interest are highlighted. The black dot represents the status quo. The black arrows indicate the direction of preference, that is, the lower total costs, the better, with an ideal policy having the lowest total costs in Germany and the Netherlands.

**Fig 5:**
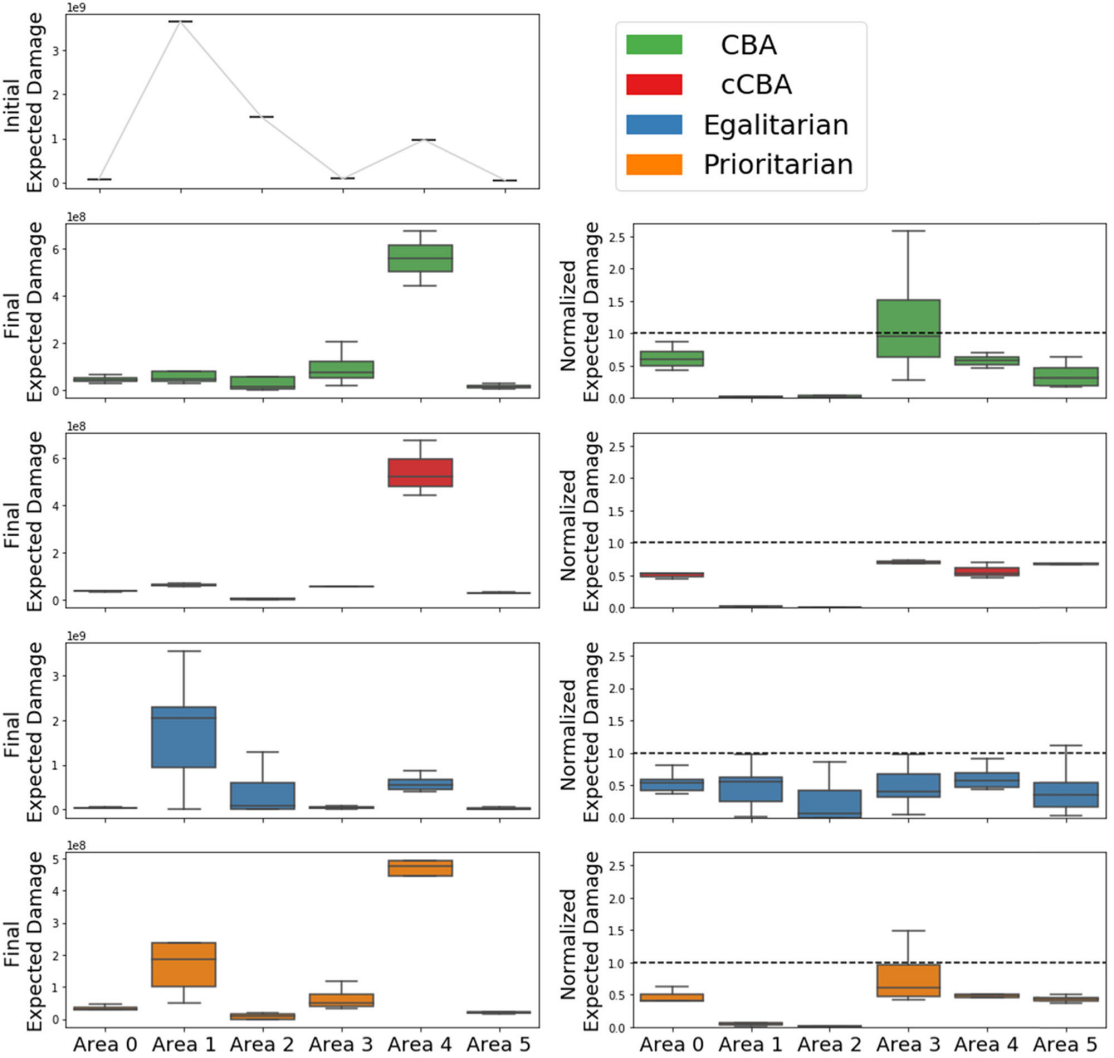
Left column: Initial risks (first row) and final risks (from second to last row) for each problem formulation. Right column: final risk normalized over initial risk values for each problem formulation. The dotted lines represent a value equal to one, that is, the status quo. Each color represents a problem formulation as indicated in the legend.

**Fig 6:**
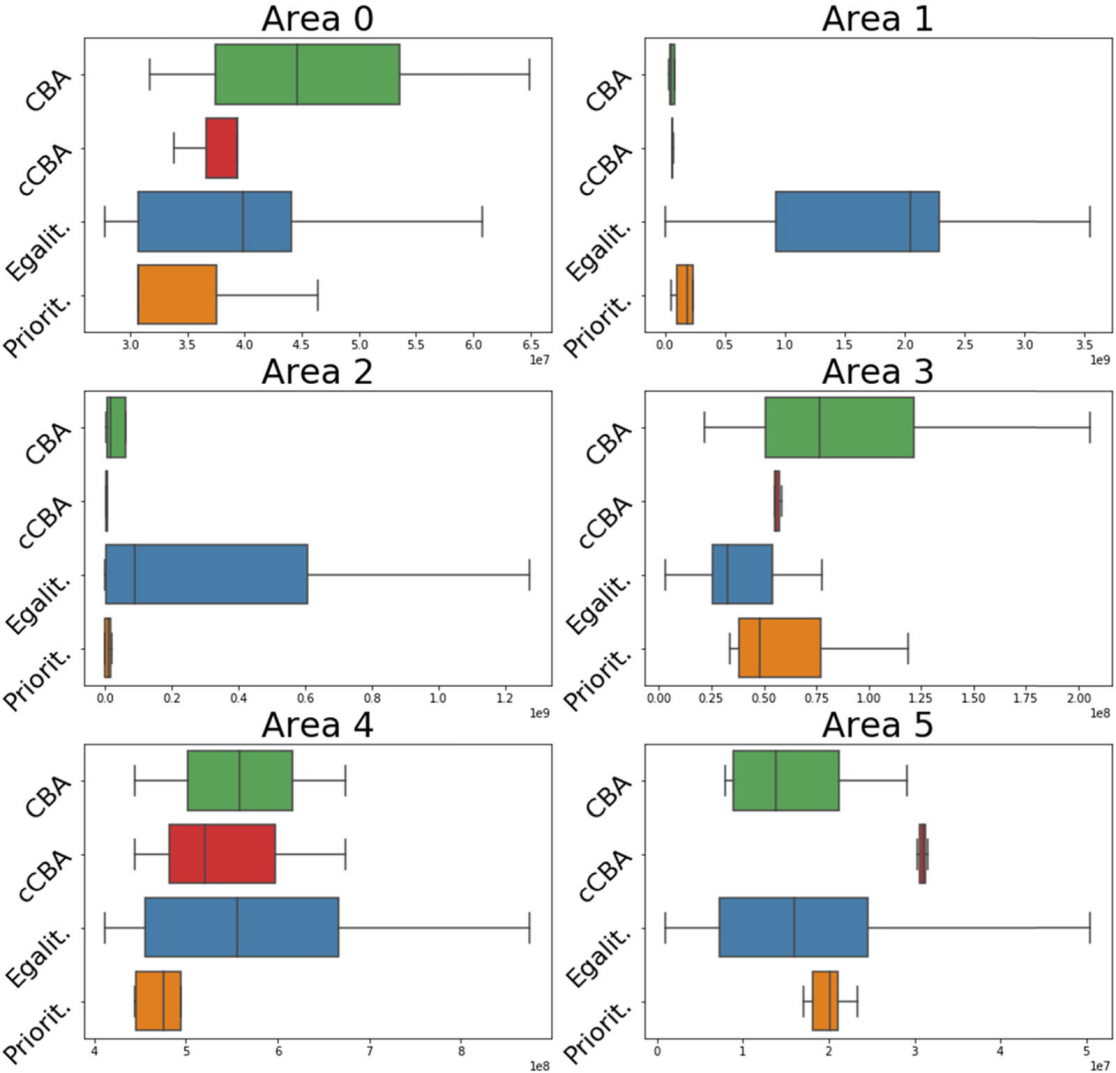
Boxplots of final risk levels across problem formulations for all geographical areas. Each color represents a problem formulation as reported on the *y*‐axis.

**Fig 7:**
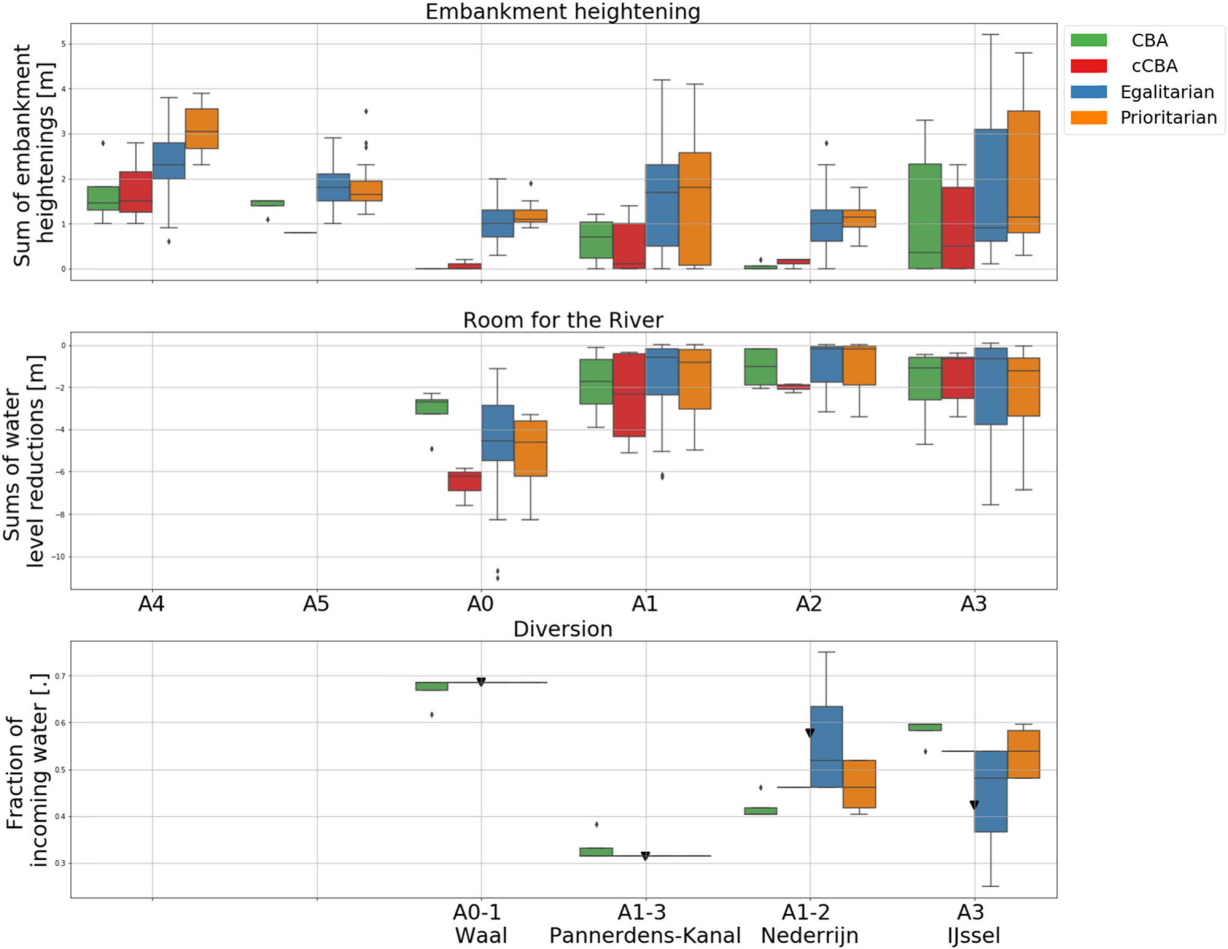
Boxplots of decision variables of the Pareto approximate sets where each color represents a problem formulation as indicated in the legend. The first row shows levels of embankment heightening. The second row shows the degree of water level lowering obtained from making room for the river. In the first and second rows, German areas are reported first. The third row shows the fraction of incoming water discharged to each branch: the Waal (affecting areas A0 and A1), the Pannerdens Canal (affecting areas A1, A2, and A3), the Nederrijn (affecting areas A1, A2), and the IJssel (affecting area A3). The default distribution (i.e., no policy change) is shown by the black triangle.

**Fig 8:**
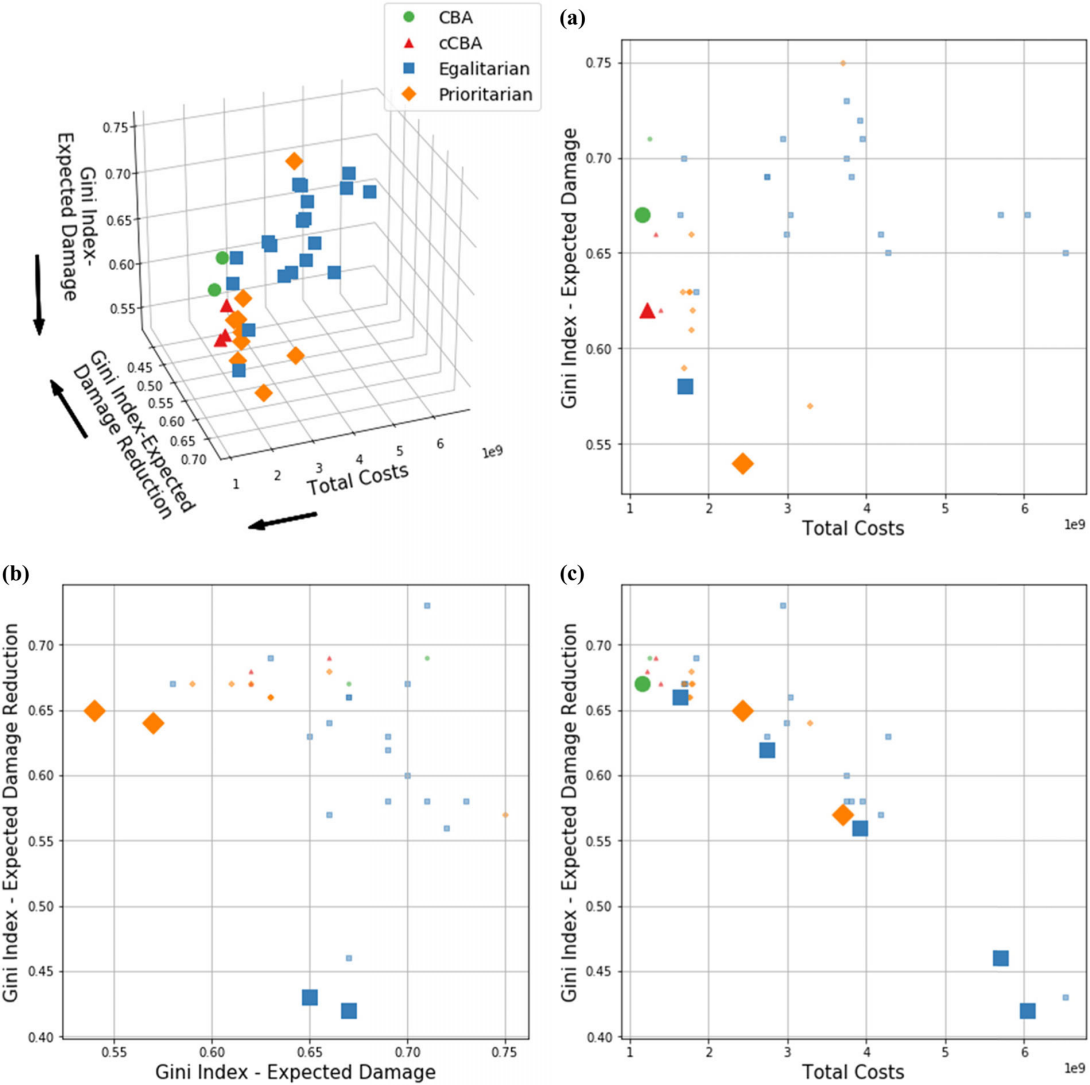
Performance of policies in terms of total costs, the Gini index for final levels of expected annual damages and the Gini index for expected annual damage reduction with respect to the staus quo. The top‐right panel shows performances in the 3D space and the direction of preference of each decision criterion, whereas the other panels show each a different 2D projection of the 3D panel.

First, results are shown based on decision objectives. In particular, policies’ performances are assessed in terms of aggregated total costs, investment costs, and final risk levels of the two countries (Fig. 4) and final risk levels of each geographic area (Figs. 5 and 6). Second, policies are shown in terms of decision variables for each geographic area (Fig. 7). Last, the Gini index and total costs for the two countries are compared (Fig. 8).

Fig. 4 shows total costs, investment costs, and final risks of the two countries for each problem formulation. It is worth stressing that the *CBA* and *cCBA* are based on optimizing total costs only, thus, a Pareto front can be clearly recognized. These problem formulations reach very low total costs, implying very low investment costs and aggregated final risks.

Policies from *egalitarian* and *prioritarian* lead to higher total costs. Interestingly, *egalitarian* requires higher investment costs and results in larger final expected damage, whereas *prioritarian* only requires higher investment costs with expected damage levels being comparable to those reached by the formulations based on cost‐benefit analysis. Finally, it is found that an improvement with respect to the status quo is reached by all problem formulations except for *egalitarian*, where some policies result in higher total costs than the status quo, because the investments are higher than the achieved aggregated risk reduction.

Fig. 5 shows final risk levels both in absolute terms as well as normalized over the initial value. In the case of normalized risk levels, crossing the dotted horizontal line means a risk increase with respect to the initial level. The figure shows the results across geographical areas and for each problem formulation. Fig. 6 shows complementary results, where absolute final risk levels are shown across problem formulations and for each area.

Interestingly, results on the left column of Fig. 5 show that only *egalitarian* maintains the same ranking of risk levels across geographical areas with respect to the initial situation. This is in line with the definition of this formulation, where risk reduction is distributed equally across areas. In the other problem formulations, the area with the largest final risk is area 4, which is, however, the area with the third largest risk in the initial situation. Areas 1 and 2, which are those with the largest initial risk, have their risk decreased. This especially occurs in the *CBA* and *cCBA*, meaning that a unit of investment cost provided the largest risk reduction in these areas. *Prioritarian* is in line with these two formulations but provides less risk reduction to area 1. At the same time, however, as can be seen from Fig. 6, *prioritarian* is the best performer of all formulations for area 4, which is the area where final risk is the largest.

The right column of Fig. 5 shows a risk increase in area 3 for*CBA* in about 50% of the cases with respect to the status quo. This means that a risk reduction for the system as a whole is achieved at the expense of one area. This is a direct consequence of the aggregated cost efficiency nature of this formulation, which is known as the *aggregation worry* (Hayenhjelm, 2012). Related to this, *CBA* is also the worst performer in area 3 (see Fig. 6). Although less frequently, also *prioritarian* can lead to risk increase in area 3. However, this only occurs after re‐evaluating under the larger sample (as shown by Fig. 11 in the Supporting information) and, therefore, it is not due to the way the formulation is defined. Formulation *cCBA* is the only one that always leads to an improved situation for all areas. This is in line with the definition of this formulation, which is still met after the re‐evaluation under the larger sample. As can be seen from Fig. 6, *cCBA* can however perform poorly for low risk areas like area 5. Finally, normalized risk levels of *egalitarian* suggest a comparable distribution of benefits among all areas, which, however, can lead to very poor performances in high risk areas, as can be seen from the performance in areas 1 and 2 in Fig. 6.

To sum up, *CBA* performs very well for high risk areas at the expense of one other area, where risk increases. The *cCBA* formulation brings a net benefit to all areas, but compared to the other formulations it can perform poorly for initially low risk areas. *Egalitarian* distributes risk reduction equally across all areas, and in so doing it performs poorly for all initially high‐risk areas. *Prioritarian* performs similarly to *CBA* and *cCBA* in terms of allocation of risk reduction, but it is never the worst performer, as it is instead the case for both *CBA* and *cCBA*. In particular, focusing on final risk at area 4 under *prioritarian*, although this is the highest among all areas (Fig. 5), it is still the lowest when compared to what result from the other problem formulations (Fig. 6).

Fig. 7 shows the identified optimal policies in terms of required interventions for all problem formulations. Changing the discharge distribution affects more than one area, therefore, in Fig. 7, the name of the river branch is specified along with the affected areas. From the top to the bottom, rows show boxplots expressing the sum of the embankment heightening, the sum of the water level lowering due to making Room for the River and the fraction of the discharge diverted to each branch. It is worth stressing that, at the second bifurcation, similar distributions may imply quite different discharge into the distributaries, being a distribution defined as the fraction of incoming water, which of course depends on the discharge distribution at the first bifurcation. Finally, at each bifurcation point, decisions on the fraction of water sent to each branch are complementary, with their sum being always equal to one.

In terms of raising embankments, *egalitarian* and *prioritarian* lead to higher embankments everywhere. Interestingly, *cCBA* leads to lower German embankments in area 5 which is for the sake of protecting the downstream area 3 along the IJssel River. In terms of discharge distribution, *CBA* supports sending slightly less water to the Waal than what is currently done and, consequently, more into the other branches. All remaining problem formulations keep the current discharge distribution at the first bifurcation point. At the second bifurcation point, most of the water is sent to the IJssel, with the Nederrijn having its discharges substantially reduced. Yet, there are some differences across problem formulations.

Overall, *cCBA* is more conservative in terms of discharge distribution (i.e., closer to the status quo) than *CBA*. This reveals how important discharge distribution policies are in regulating risk levels across the system and in guaranteeing that none of the areas has its risk disproportionately increased. *Egalitarian* and *prioritarian* imply similar embankment heightening and Room for the River projects along areas 1 and 2. In *prioritarian*, however, the Nederrijn receives less water, resulting in an overall higher protection level than in *egalitarian* (as can be also seen in Fig. 6).

Fig. 8 shows the performance of the policies in terms of systems’ total costs (i.e., sum of total costs in Germany and the Netherlands) and two different evaluations of the Gini index. The Gini index is evaluated in terms of expected damage and expected damage reduction. In this figure, only those policies which do not increase risk in any area are considered.

Policies are shown in the 3D space and in the three 2D spaces, to highlight the Pareto front between each pair of decision criteria (i.e., plots *a, b, c*). An ideal policy would be found in the bottom‐left corner of each of these plots. A trade‐off between total cost (i.e., efficiency) and the Gini‐index scores (i.e., equal distribution of benefits) emerges, in both evaluations of the Gini index (i.e., plots *a, b*). The most efficient policy is obtained from *CBA*, and this is in line with the nature of the formulation. In plot (*a*), a slight increase in terms of equity can be achieved with*cCBA* and the lowest Gini can be reached with *prioritarian*.

The same results are found in plot (*b*), where, however, higher equity (low Gini) is reached by *egalitarian*. This is in accordance with the way expected damage distribution is conceptualized in the two formulations. In the case of *egalitarian*, expected damage is distributed regardless the initial levels of each area, thus, trying to achieve the most equal distribution of expected damage reduction. In contrast, *prioritarian* prioritizes investments in higher initial expected damage areas, thus, levelling the gap in terms of final levels of expected damages. This is also evident in plot (*c*), where the two evaluations of the Gini index are compared. The best performers (low Gini) in terms of expected damage reduction belong to *egalitarian*, while *prioritarian* leads to lower Gini when expected damages are considered.

## 8 DISCUSSION AND CONCLUSIONS

In the present study, we propose a decision criterion to properly account for the distribution of benefits of a given policy across geographical areas. The criterion is used to explore the policy implications of adopting alternative ethical principles in supporting flood risk management decisions. The area of application is the Lower Rhine River.

Four ethical principles are considered, each leading to a different problem formulation. The first and second problem formulations (*CBA* and *cCBA*, respectively) are based on minimizing total costs, the difference being that in *cCBA* no risk increase with respect to the status quo is allowed in any area. In the third problem formulation (*egalitarian*), risk reduction is distributed equally among areas. The fourth problem formulation *(prioritarian*) distributes risk by prioritizing areas with larger risk.

Because of the aggregation of costs and benefits, *CBA* leads to the so‐called *aggregation worry*, that is, it performs well for some areas at the expense of other areas where risk increases. Although *cCBA* overcomes this, it leads to an unbalanced risk distribution by favoring some areas at the cost of others. *Egalitarian* increases the equity of the implemented policies; however, it performs very poorly for high risk areas and, in general, it costs more and yet results in larger aggregated risk. *Prioritarian* reduces expected damages following the same allocation pattern as *CBA* and *cCBA*. It never performs as the worst formulation and it achieves the highest risk reduction in the area that is worst off compared to the other formulations. In other words, it seems to be economically efficient while limiting unbalances in benefit distribution between areas. This latter point is achieved by investing more in comparison to *CBA* and *cCBA* but at the same time spending money more wisely (from an economic viewpoint) than *egalitarian*.

Although presented in the context of flood risk management, the proposed approach is general as it improves model‐based decision support by enabling to account for risk distribution. The approach reveals otherwise hidden trade‐offs between risk reduction and risk distribution objectives, thus, broadening the spectrum of policy objectives most cost‐benefit analyses rely on. Furthermore, as it is found that the choice of *what* ought to be distributed matters, the proposed approach allows taking into account the effects of alternative distributional choices on the performance of policies and, as such, it enables policy makers to operationalize alternative ethical principles and to elicit their preferences in balancing efficiency in risk reduction and equity in risk distribution.

Finally, the proposed framework relies on a decision criterion that is defined based on area‐wide performances, that is, on changes in overall risk of flood protected areas. As these areas can generally be very large and diverse in terms of internal socioeconomic conditions, future research may focus on advancing the presented approach by adopting a finer resolution. On the one hand, this will increase the complexity of the analysis as it will require dealing with a larger number of interested parties and, therefore, of decision objectives to optimize. On the other hand, it will allow accounting for risk shifts while also taking into account the socioeconomic peculiarities of the various communities living within a given area and, therefore, differentiating between wealthy and deprived communities.

## Supporting information


**Fig. 1**. The modeling scheme (adapted from Ciullo et al. 2019).
**Fig. 2**. Hypervolume of the final set of the final set of epsilon‐dominant solutions after the seed analysis
**Fig. 3**. Epsilon‐progress and hypervolume progression against the number of function evaluations of the five optimizations of the first problem formulation.
**Fig. 4**. Epsilon‐dominant solutions after five optimizations for the first problem formulations: objectives relate to total costs in the Netherlands (Total Costs_nl, euros) and total costs in Germany (Total Costs_de, euros).
**Fig. 5**. Epsilon‐progress and hypervolume progression against the number of function evaluations of the five optimizations of the second problem formulation.
**Fig. 6**. Epsilon‐dominant solutions after five optimizations for the second problem formulations: objectives relate to total costs in the Netherlands (Total Costs_nl, euros) and total costs in Germany (Total Costs_de, euros).
**Fig. 7**. Epsilon‐progress and hypervolume progression against the number of function evaluations of the five optimizations of the third problem formulation.
**Fig. 8**. Epsilon‐dominant solutions after five optimizations for the third problem formulations: objectives relate to total costs in the Netherlands (Total Costs_nl, euros) and total costs in Germany (Total Costs_de, euros), and the distance criterion of each area.
**Fig. 9**. Epsilon‐progress and hypervolume progression against the number of function evaluations of the five optimizations of the fourth problem formulation.
**Fig. 10**. Epsilon‐dominant solutions after five optimizations for the fourth problem formulations: objectives relate to total costs in the Netherlands (Total Costs_nl) and total costs in Germany (Total Costs_de), and the distance criterion of each area.
**Fig. 11**. Comparison of the performance of the optimal policies under the reference sample and under a 250 times larger sample. The first row shows results in terms of total costs in Germany and the Netherlands.
