# Supplementary material for: Efficient or Fair? Operationalizing Ethical Principles in Flood Risk Management: A Case Study on the Dutch‐German Rhine
Source: Risk Anal. 2020 Jun 11;40(9):1844–62. doi: 10.1111/risa.13527 (PMC7540719; doi:10.1111/risa.13527)
Supplement: Supplementary file 1 — Fig. 1. The modeling scheme (adapted from Ciullo et al. 2019). Fig. 2. Hypervolume of the final set of the final set of epsilon‐dominant solutions after the seed analysis Fig. 3. Epsilon‐progress and hypervolume progression against the number of function evaluations of the five optimizations of the first problem formulation. Fig. 4. Epsilon‐dominant solutions after five optimizations for the first problem formulations: objectives relate to total costs in the Netherlands (Total Costs_nl, euros) and total costs in Germany (Total Costs_de, euros). Fig. 5. Epsilon‐progress and hypervolume progression against the number of function evaluations of the five optimizations of the second problem formulation. Fig. 6. Epsilon‐dominant solutions after five optimizations for the second problem formulations: objectives relate to total costs in the Netherlands (Total Costs_nl, euros) and total costs in Germany (Total Costs_de, euros). Fig. 7. Epsilon‐progress and hypervolume progression against the number of function evaluations of the five optimizations of the third problem formulation. Fig. 8. Epsilon‐dominant solutions after five optimizations for the third problem formulations: objectives relate to total costs in the Netherlands (Total Costs_nl, euros) and total costs in Germany (Total Costs_de, euros), and the distance criterion of each area. Fig. 9. Epsilon‐progress and hypervolume progression against the number of function evaluations of the five optimizations of the fourth problem formulation. Fig. 10. Epsilon‐dominant solutions after five optimizations for the fourth problem formulations: objectives relate to total costs in the Netherlands (Total Costs_nl) and total costs in Germany (Total Costs_de), and the distance criterion of each area. Fig. 11. Comparison of the performance of the optimal policies under the reference sample and under a 250 times larger sample. The first row shows results in terms of total costs in Germany and the Netherlands. [file RISA-40-1844-s001.docx]

SUPPLEMENTARY MATERIAL

In the present document, we provide information about the simulation model, the many-objectives evolutionary algorithm ε-NSGAII and report the results from the optimizations relative to each problem formulation.

# THE MODELLING CHAIN

The modelling chain is adapted from Ciullo, de Bruijn, Kwakkel, and Klijn (2019) and is illustrated in Fig. 1. Here we provide more information about the modelling steps not covered in the main manuscript: pre-processing, event generation and event simulation.

## PRE-PROCESSING

The pre-processing step involves (1) calibration of the discharge routing scheme and (2) calculation of critical water levels at all locations. Flood routing is modelled with a Muskingum scheme (Todini, 2007) which is applied to all subsequent potential breach locations. The scheme is benchmarked against the results of a *SOBEK* model calibrated on the case study by following the method of least-squares as in Karahan (2012) . Critical water levels are defined as water levels at which failure of the embankment is triggered. At each location, they are calculated as those having a probability of occurrence equal to the standards for flood protection.

## 1.2 EVENT GENERATION

The event generation step requires the sampling of (1) upstream flood discharge and (2) flood risk reduction measures. Maximum upstream flood discharges are generated following the Generalized Extreme Value distribution Type I, i.e. a Gumbel distribution, proposed in De Bruijn, Diermanse, and Beckers (2014), and Diermanse, De Bruijn, Beckers, and Kramer (2014). A flood hydrograph is then generated by multiplying the sampled maximum discharge with one of the plausible normalized hydrographs derived by the *GRADE* project (Generator of Rainfall and Discharge Extremes) (Hegnauer, Beersma, van den Boogaard, Buishand, & Passchier, 2014).

## 1.3 EVENT SIMULATION

Once an event is generated, the flood hydrograph propagates through the river. At each location, (1) the incoming discharge is translated into water levels through a stage-discharge relationship and (2) embankment failure is evaluated by comparing the water levels in the river with the *critical water levels*. When the latter are exceeded a breach is triggered. The discharge through the breach is simulated using a weir formula and the downstream discharge is reduced accordingly. The breach growth is simulated assuming exponential growth, with a maximum breach width of 150 meters which is reached after 6 hours.


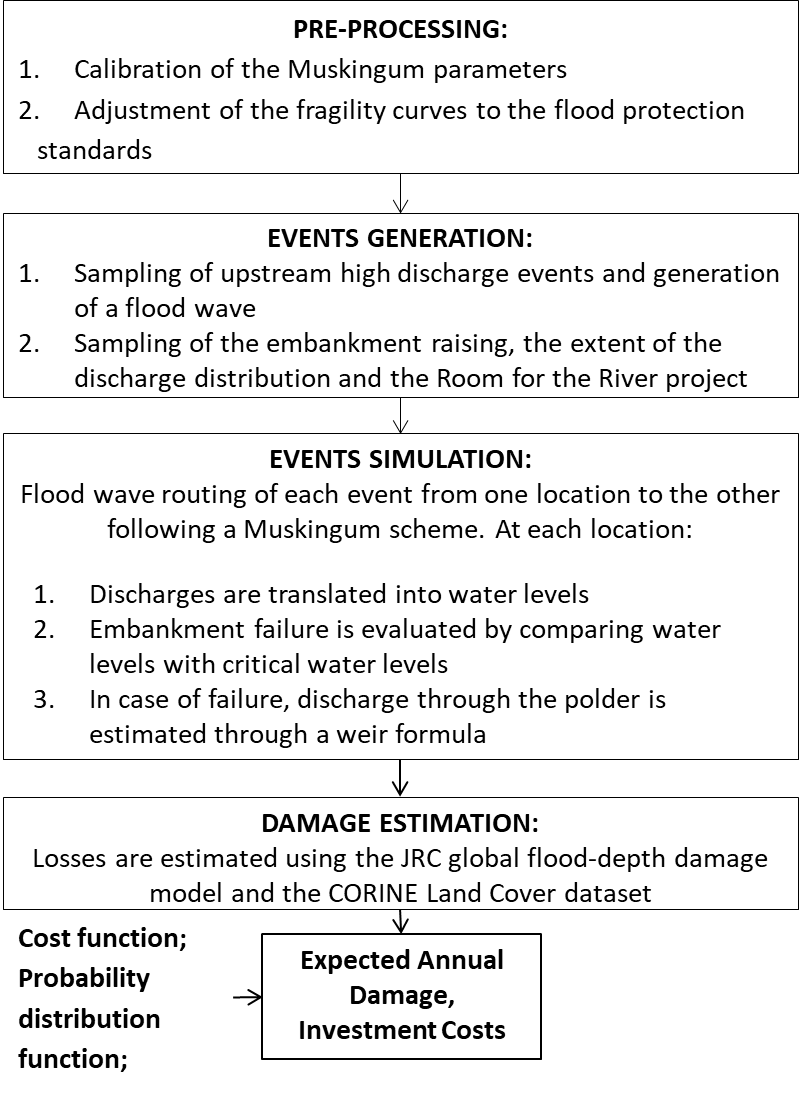


Fig. 1. The modeling scheme (adapted from Ciullo et al. 2019).

# OPTIMIZATION OF THE PROBLEM FORMULATIONS

## 2.1 ε-NSGAII

ε-NSGAII is a state of the art generational many-objective evolutionary algorithm. It builds on NSGAII (Deb, Pratap, Agarwal, & Meyarivan, 2002) by including features like epsilon archiving, auto-adaptive sizing, and conditional rebuilding of the population of solutions.

Epsilon-archiving means that an approximate representation of the observed Pareto front is archived along the search process, with ε determining the resolution of the approximation (Laumanns, Thiele, Deb, & Zitzler, 2002). In particular, a multi-dimensional grid of size ε is imposed on the objective space, and per grid cell a single solution is maintained in the archive. The smaller the ε values, the finer the grid and the larger the number of solutions. ε–dominance thus allows the user to specify the precision with which objectives are quantified. The resolution must be chosen well. Using a very small resolution may lead to a disproportionally large number of solutions undermining the computational viability of the approach, while using a very course resolution may prevent reliable identification of the Pareto front. Auto-adaptive sizing of the population of solutions means that the size of the population is adapted in response to the size of the archive. The conditional rebuilding of the population is triggered if either the desired population size is different from the actual population size, or if the observed progress has stalled. A new population is then created, starting from the existing archive (Reed, Hadka, Herman, Kasprzyk, & Kollat, 2013). In various comparisons, ε-NSGAII has performed very well (Hadka & Reed, 2013).

The choice for ε-NSGAII in this paper is a pragmatic one: source code is readily available, and it is easy to parallelize for speed up purposes. The success of any MOEA in finding an approximation of the Pareto front is measured by the convergence (the evolution of the Pareto front) and diversity (degree of distribution of the solutions over the entire Pareto front) of the solutions (Reed, Hadka, Herman, Kasprzyk & Kollat, 2013). To quantify these, we use ε-progress (Hadka & Reed, 2013) and hypervolume (Zitzler, Thiele, Laumanns, Fonseca, & da Fonseca, 2003). ε-Progress (Hadka & Reed, 2013) indicates whether a new solution is added to the ε-archive that is in a new grid cell. Hypervolume is the volume of objective space dominated by a given set of solutions. By calculating the hypervolume of the archive for each iteration, we can track progress.

Since MOEAs rely on the random generation of the initial population and because of the probabilistic nature of the evolutionary operators, it is good practice to perform a seed analysis. Thus, each problem formulation is solved *five* times. Then, for each problem formulation, a final set of ε–dominant solutions are evaluated across the five sets of solutions. For each MOEA search, the first two problem formulations are evaluated 100.000 times, the third 300.000 times and the fourth 200.000 times, for a total of 3 million evaluations.

The hypervolume values of the final set of ε–dominant solutions for the four problem formulations are shown in Fig. 1. Figs. from 2 to 9, instead, show results of each optimization in the seed analysis in terms of epsilon-progress and hypervolume progression against the number of function evaluations as well as the final Pareto front.


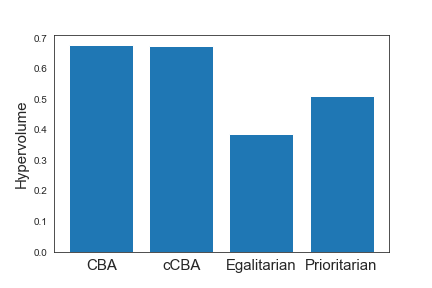


Fig. 2. Hypervolume of the final set of the final set of epsilon-dominant solutions after the seed analysis

## 2.2 FIRST PROBLEM FORMULATION - *CBA*:


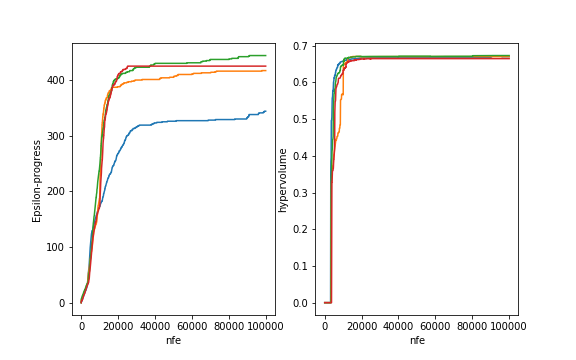


Fig. 3. Epsilon-progress and hypervolume progression against the number of function evaluations of the five optimizations of the first problem formulation.


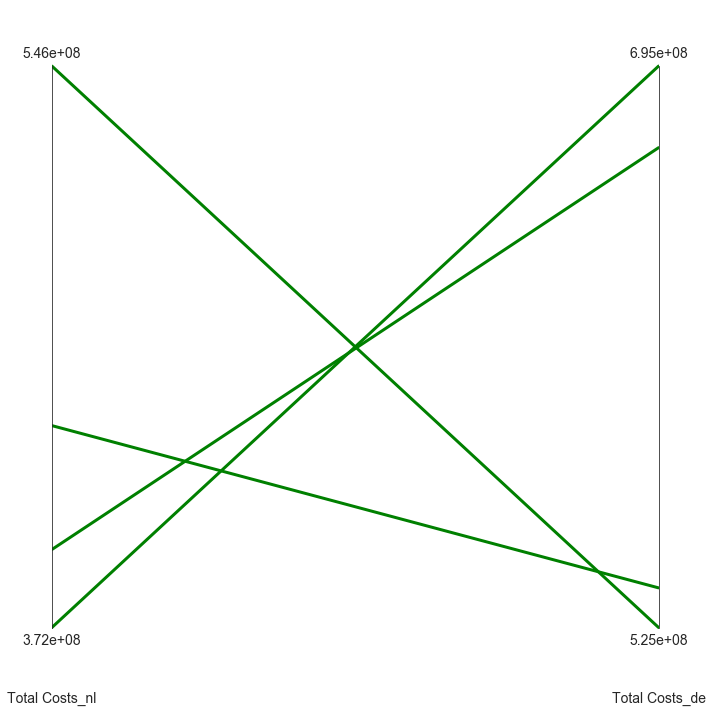


Fig. 4. Epsilon-dominant solutions after five optimizations for the first problem formulations: objectives relate to total costs in the Netherlands (Total Costs_nl, euros) and total costs in Germany (Total Costs_de, euros).

## 2.3 SECOND PROBLEM FORMULATION - *cCBA*:


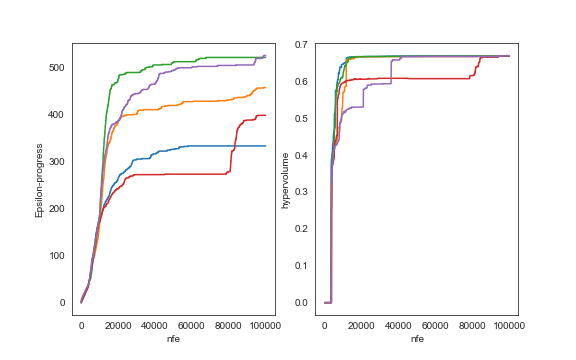


Fig. 5. Epsilon-progress and hypervolume progression against the number of function evaluations of the five optimizations of the second problem formulation.


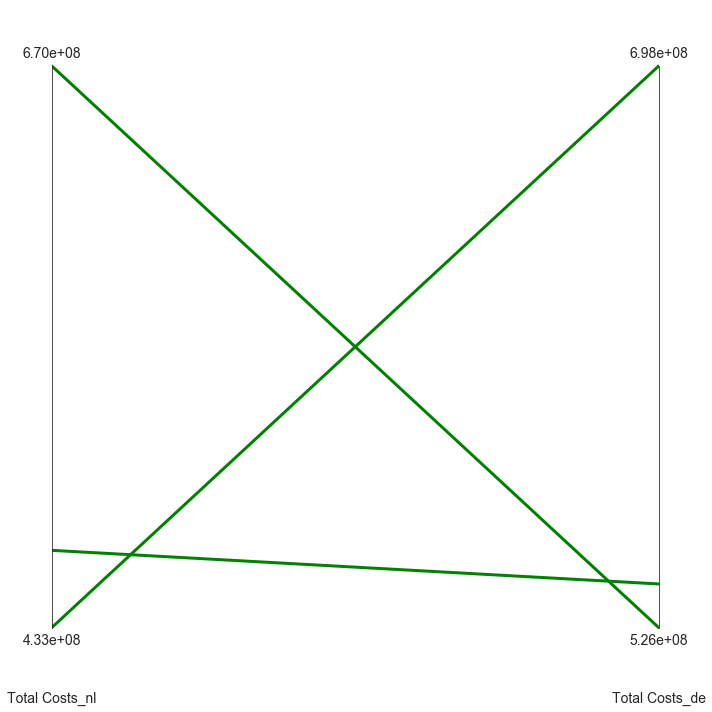


Fig. 6. Epsilon-dominant solutions after five optimizations for the second problem formulations: objectives relate to total costs in the Netherlands (Total Costs_nl, euros) and total costs in Germany (Total Costs_de, euros).

## 2.4 THIRD PROBLEM FORMULATION - *EGALITARIAN*:


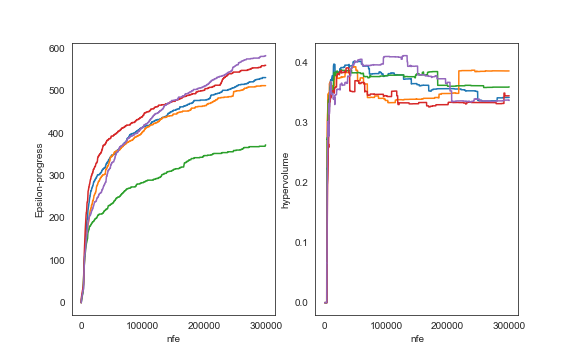


Fig. 7. Epsilon-progress and hypervolume progression against the number of function evaluations of the five optimizations of the third problem formulation.


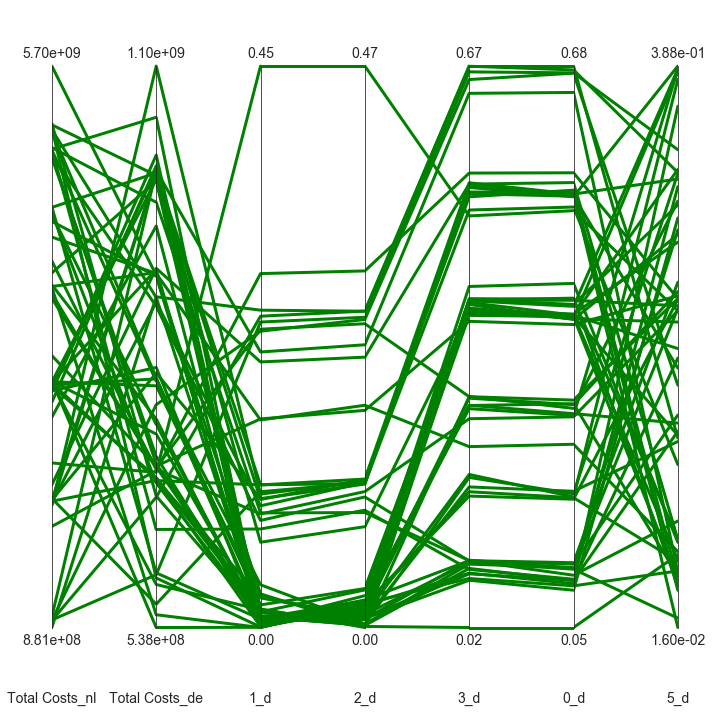


Fig. 8. Epsilon-dominant solutions after five optimizations for the third problem formulations: objectives relate to total costs in the Netherlands (Total Costs_nl, euros) and total costs in Germany (Total Costs_de, euros), and the distance criterion of each area.

## 2.5 FOURTH PROBLEM FORMULATION - *PRIORITARIAN*:


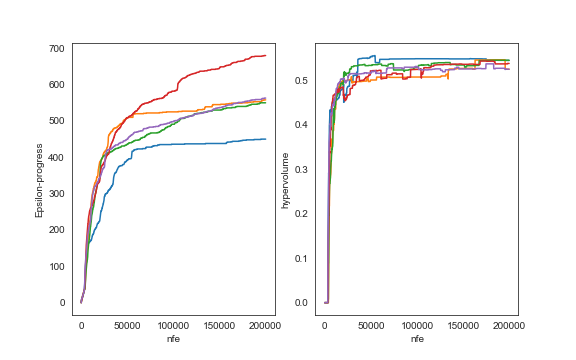


Fig. 9. Epsilon-progress and hypervolume progression against the number of function evaluations of the five optimizations of the fourth problem formulation.


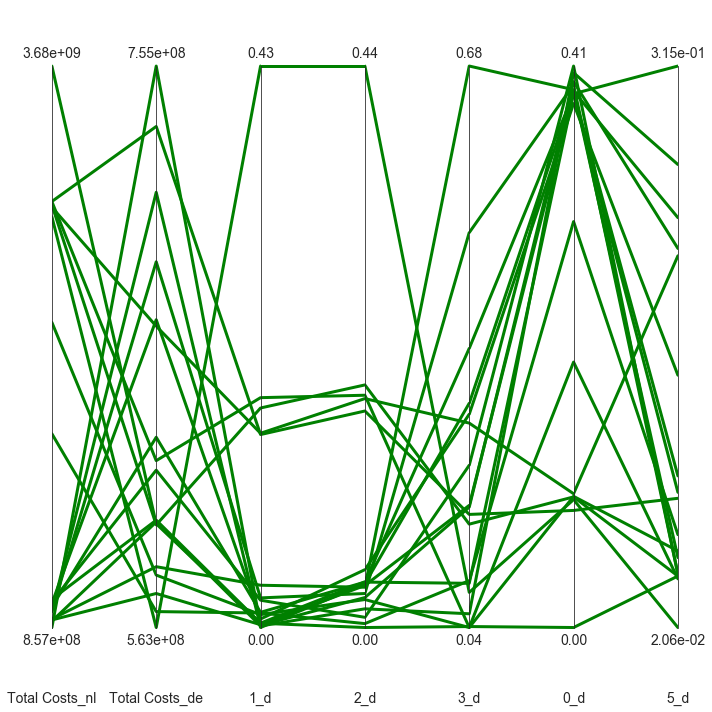


Fig. 10. Epsilon-dominant solutions after five optimizations for the fourth problem formulations: objectives relate to total costs in the Netherlands (Total Costs_nl) and total costs in Germany (Total Costs_de), and the distance criterion of each area.

# RE-EVALUATION OF THE OPTIMAL POLICIES UNDER A LARGER SAMPLE

Fig. 11 shows a comparison of the performance of the optimal policies found with NSGAII-ε under the reference sample of 10 high-flood waves and a larger sample of 2500 high-flood waves. The figure shows that total costs in the Netherlands and expected damages of areas 1 and 2 are very close to the bisector, which means that the small set can be considered representative of the larger set of flood waves. Total costs in Germany and the expected damages of areas 3 and 4 are slightly underestimated by the reference sample. Expected damages of area 5 are significantly underestimated by the reference sample, as it is for area 0, where, in addition, very poor correlation is found.

In area 3, one CBA policy (policy P) leads to an increase of risk with respect to the status quo (see main text for further comments). When looking at total costs for the two countries, some policies found under the egalitarian formulation lead to an increase in total costs with respect to the status quo, indicating that the egalitarian formulation is not economically efficient (see main text for further comments).


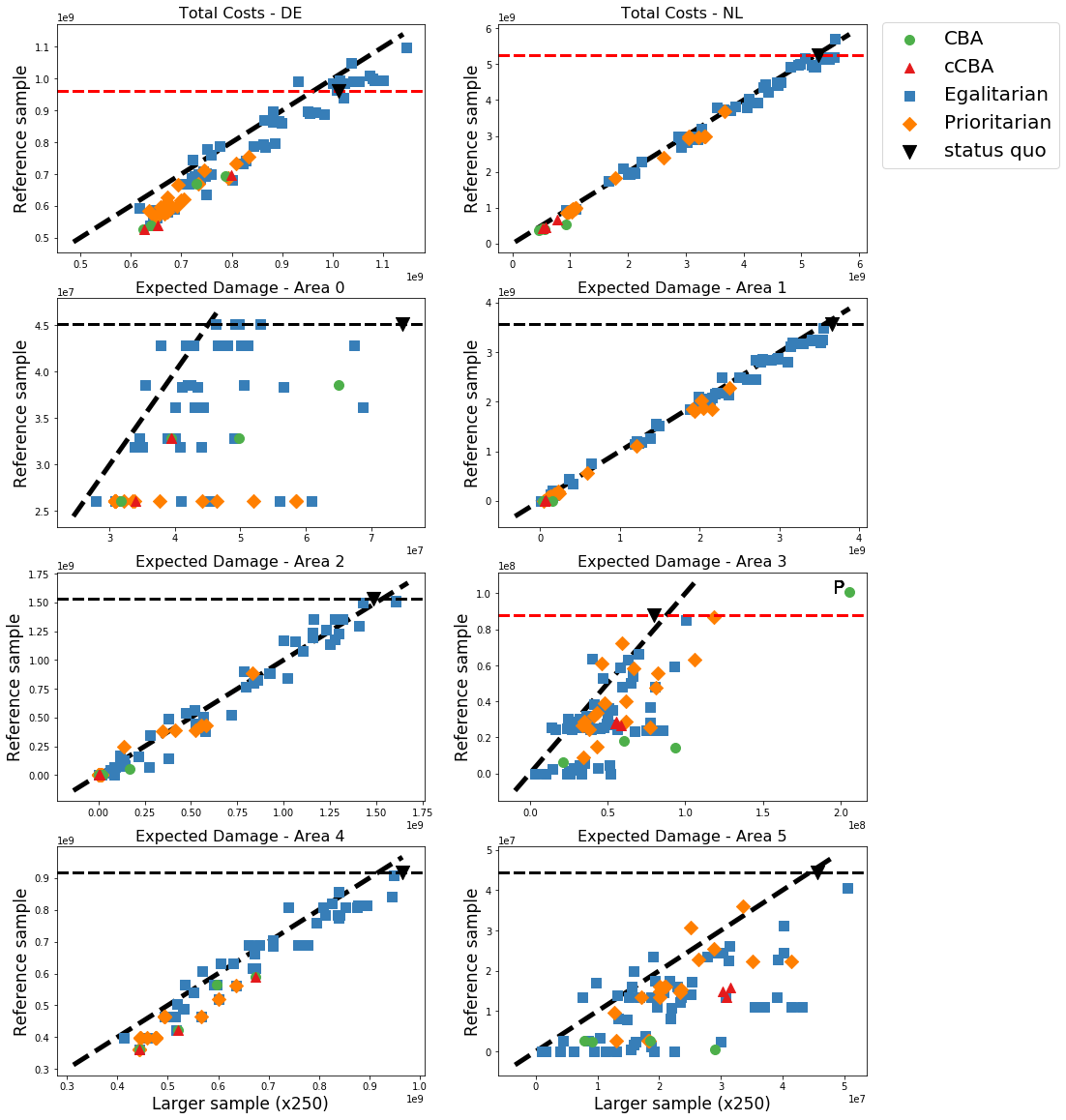


Fig. 11. Comparison of the performance of the optimal policies under the reference sample and under a 250 times larger sample. The first row shows results in terms of total costs in Germany and the Netherlands. The other rows show results in terms of risk for each flood-protected area. Problem formulations follow colours and marks indicated in the legend. In each panel, the dotted horizontal line represents the performance of the status quo under the reference sample. Such line is black when none of the optimal policies underperform with respect to the status quo, and red otherwise.

# REFERENCES

De Bruijn, K. M. De, Diermanse, F. L. M., & Beckers, J. V. L. (2014). An advanced method for flood risk analysis in river deltas , applied to societal flood fatality risk in the Netherlands. *Natural Hazards and Earth System Sciences*, *14*(10), 2767–2781. https://doi.org/10.5194/nhess-14-2767-2014

Ciullo, A., de Bruijn, K. M., Kwakkel, J. H., & Klijn, F. (2019). Accounting for the uncertain effects of hydraulic interactions in optimising embankments heights: Proof of principle for the IJssel River. *Journal of Flood Risk Management*, (December 2018), e12532. https://doi.org/10.1111/jfr3.12532

Deb, K., Pratap, A., Agarwal, S., & Meyarivan, T. (2002). A fast and elitist multiobjective genetic alghoritm: NSGA-II. *IEEE Transactions on Evolutionary Computation*, *6*(2), 182–197.

Diermanse, F. L. M., De Bruijn, K. M., Beckers, J. V. L., & Kramer, N. L. (2014). Importance sampling for efficient modelling of hydraulic loads in the Rhine–Meuse delta. *Stochastic Environmental Research and Risk Assessment*, *29*(3), 637–652. https://doi.org/10.1007/s00477-014-0921-4

Hadka, D., & Reed, P. (2013). Borg: An Auto-Adaptive Many-Objective Evolutionary Computing Framework. *Evolutionary Computation*, *21*(2), 231–259.

Hegnauer, M., Beersma, J. J., van den Boogaard, H. F. P., Buishand, T. A., & Passchier, R. H. (2014). *Generator of Rainfall and Discharge Extremes (GRADE) for the Rhine and Meuse basins*.

Karahan, H. (2012). Predicting Muskingum Flood Routing Parameters Using Spreadsheets. *Computer Applications in Engineering Education*, *20*(2), 280–286. https://doi.org/10.1002/cae.20394

Laumanns, M., Thiele, L., Deb, K., & Zitzler, E. (2002). Combining Convergence and Diversity in Evolutionary Multiobjective Optimization. *Evolutionary Computation*, *10*(3), 263–282. https://doi.org/10.1162/106365602760234108

Reed, P. M., Hadka, D., Herman, J. D., Kasprzyk, J. R., & Kollat, J. B. (2013). Advances in Water Resources Evolutionary multiobjective optimization in water resources : The past , present , and future. *Advances in Water Resources*, *51*, 438–456. https://doi.org/10.1016/j.advwatres.2012.01.005

Todini, E. (2007). A mass conservative and water storage consistent variable parameter Muskingum-Cunge approach. *Hydrology and Earth System Sciences*, *11*(5), 1645–1659. https://doi.org/10.5194/hess-11-1645-2007

Zitzler, E., Thiele, L., Laumanns, M., Fonseca, C. M., & da Fonseca, V. G. (2003). Performance assessment of multiobjective optimizers: an analysis and review. *IEEE Transactions on Evolutionary Computation*, *7*(2), 117–132. https://doi.org/10.1109/TEVC.2003.810758
